# Compressed Air

**Published:** 1885-02

**Authors:** 


*Compressed Air.*—Masini propounds to substitute the inhalations of compressed oxygenized air to the simple inhalations of oxygen in croup, trusting to the mechanical action exercised by the compressed air on the narrow walls of the larynx, and on the chemical action of the blood due to an increase of the inspired oxygen. He uses the Waldenburg apparatus, adding the sufficient quantity of oxygen. The applications must be of long duration and repeated four to fives times a day. This treatment does not exclude other remedies, and by prolonging the course of the disease, gains time for the surgeon to perform tracheotomy under favorable conditions.—*Italia Medica.*

